# Clinical and aetiologic profile of patients with chronic urticaria at the outpatient clinic of a tertiary hospital in Lagos, Nigeria: a cross-sectional observational study

**DOI:** 10.11604/pamj.2021.40.141.27655

**Published:** 2021-11-05

**Authors:** Shakirat Gold-Olufadi, Olusola Ayanlowo, Ayesha Omolara Akinkugbe, Erere Otrofanowei

**Affiliations:** 1 University College Hospital, Oyo State, Ibadan, Nigeria,; 2 University of Lagos, Lagos University Teaching Hospital, Lagos, Nigeria

**Keywords:** Chronic urticaria, aetiology, precipitants

## Abstract

Chronic urticaria is a condition characterized by recurrent wheals associated with itching lasting longer than six weeks. The condition tends to run a chronic course with significant morbidity. Several factors have been explored in the cause, yet the aetiology is still uncertain. We documented the clinical profile and assessed the possible aetiologic factors in patients with chronic urticaria. Clinical profile of sixty consecutive patients with chronic spontaneous urticaria was assessed with possible precipitants identified using a structured questionnaire. The complete blood count with erythrocyte sedimentation rate, antithyroid peroxidase (antiTPO), hepatitis B surface antigen (HBsAg), antihepatitis C virus screening (AntiHCV), stool for ova and parasites and helicobacter pylori were carried out. Controls without a history of urticaria were recruited in a 1: 1 ratio for stool for parasites and H. pylori because of the high prevalence rates reported in this environment. Data analysis was carried out with Statistical Packages for Social Sciences (SPSS) version 22. Sixty patients with chronic urticaria and sixty age and sex matched controls were enrolled in the study. Females presented more often with urticaria with a M: F ratio of 1: 2.5. The mean age of onset of urticaria was in the third decade of life. Precipitants were identified in close to a third of patients. The investigated antigenic aetiology was not found to play a role. The presentation and clinical profile of patients in this environment is similar to previously documented studies. History still remains the cornerstone of management as precipitants and triggers may be identified. Management of patients should therefore be individualized.

## Introduction

Chronic urticaria refers to the recurrent appearance of spontaneous usually transient wheals associated with itching lasting longer than six weeks [1]. The condition tends to run a chronic course especially because the aetiology is not identified in most cases. Factors implicated in the aetiology include infections, infestations, food allergies, and autoimmunity amongst others with several classified as idiopathic [2]. Several studies have highlighted the clinical profile of chronic urticaria patients in the western world and the aim of this cross-sectional study was to add to the available knowledge with regards to the profile and possible aetiological factors of patients with chronic urticaria in Nigeria.

**Objectives:** the objectives of the study were to assess the clinical and demographic profile of patients with chronic urticaria attending dermatology outpatient clinic of Lagos University Teaching Hospital (LUTH), Lagos and to outline the possible aetiologic factors in patients with chronic urticaria.

## Methods

This study was carried out at Dermatology Outpatient Clinic of Lagos University Teaching Hospital (LUTH), Idi Araba Lagos to outline the clinical profile of patients with chronic urticaria. LUTH is a government hospital and the larger of the two teaching hospitals in the state that provides multidisciplinary tertiary care services to the inhabitants of Lagos and its surrounding states. Lagos State is unarguably the most economically important state in the nation and the former capital of Nigeria. The clinic attends to an average of about one hundred and fifty (150) patients weekly and a third of these are newly referred dermatology patients.

Ethical approval was granted by the hospital after thorough appraisal of study design and methodology by the health research and ethics committee of LUTH prior to commencement of recruitment into the study (ADM/DCST/HREC/APP/1249). The study was descriptive, and the procedures associated (blood draws) were procedures that are routinely conducted during standard clinical practice and risk exposures were minimal. Notwithstanding, informed consent was obtained from all participants without coercion or undue influence, and confidentiality, the right to withhold participation or withdraw at any time was communicated clearly. No cost related to the study was borne by participants. All data was de-identified using study identification numbers, and personal data to enable follow up and tracking of participation was held securely. The inclusion criteria for the study were individuals who had urticaria with or without angioedema with symptoms lasting greater than six weeks. Pregnant women and individuals with known co-morbidities like hypertension, diabetes mellitus, heart failure, malignancies, chronic liver disease and chronic renal failure were excluded from the study.

All consecutive patients who presented with features of chronic urticaria (either on examination at the clinic or with history suggestive of chronic urticaria supported with pictures) over a period of one year (May 2017 to April 2018) were recruited into this study. Overall, sixty patients with chronic urticaria with sixty age and sex matched controls were recruited for specific tests after an informed consent had been obtained from all participants.

The following data were collected in patients aged 15 years and above: demographic characteristics, duration of urticaria, history of angioedema, associated symptoms with urticaria, areas affected by the urticaria wheals, aggravating factors, history suggestive of physical urticaria, history of atopy, family history of urticaria in first degree relatives and previous treatment prior to presentation using an interviewer administered questionnaire. Clinical examination was performed to assess for dermographism in both patients with chronic urticaria and controls. Investigations done are as follows; complete blood count (CBC) with an erythrocyte sedimentation rate (ESR), hepatitis B surface antigen screening (HBsAg), anti hepatitis C virus (AntiHCV) screening, anti thyroid peroxidase (TPO), stool for ova and parasite, stool for helicobacter pylori antigen (H. pylori Ag). HBsAg, AntiHCV screening and stool tests for H. pylori, ova and parasites were assessed in both patients and controls because of the high prevalence of these in the tropics. The CBC analyses were performed on the Mindray BC-3200 series auto haematology analyser, the ESR was done with the Westergren method while the HBsAg and AntiHCV were carried out using a rapid immunochromatographic assay method. Stool for H. pylori antigen was performed with the enzyme immunoassay method while stool for ova and parasite was carried out using wet preparation and concentration technique with formol ether.

Statistical analysis was carried out with Statistical Package for Social Sciences version 22.0 (SPSS Chicago Inc. Illinois USA). Continuous variables were expressed as means and standard deviation while categorical variables were expressed as percentages. Charts were also used in presenting percentages and proportions. The student´s t-test was used to compare means while chi square test was used for comparison of percentages. Statistical significance was set at p value <0.05.

## Results

Sixty-eight patients were initially recruited but six were excluded because they were found to have acute urticaria and two patients declined from participating in the study. Sixty patients with chronic urticaria and sixty age and sex matched controls were enrolled in the study. There were no missing data as all the patients consented and were present till the end of the study. The study questionnaire was interviewer administered and all parameters were cross-checked twice.

**Figure 1:**
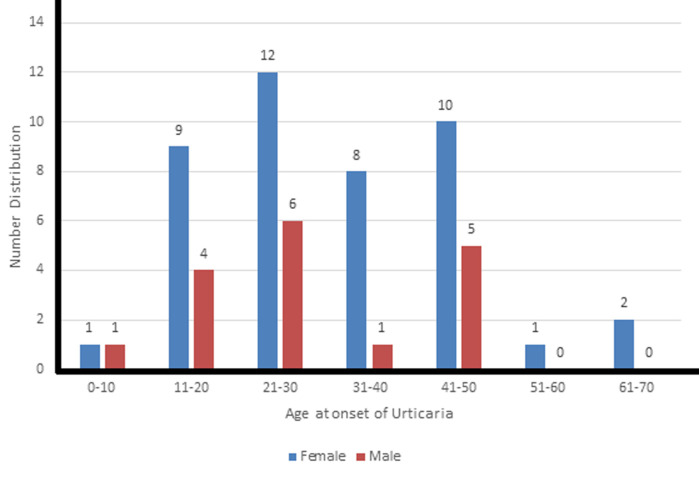
distribution of urticaria by age at onset and gender

**Demographics:** the mean age of cases (at presentation) and controls was 37.1 ± 13.7 and 36.7 ± 12.9 respectively (p = 0.88) with a female predominance. The mean age at onset of urticaria was 31.1 ± 13.3 years (females: 32.7±13.7, males: 27.3 ± 11.7, p = 0.16) and peak age at onset was in the third decade of life for both genders (Figure 1). A family history of urticaria in a first degree relative was documented in 30% (18) of patients. The mean age at onset of urticaria did not differ significantly between those with (29.4 ± 14.6) and without (31.6 ± 12.9) a family history (p = 0.57). The frequency of a positive family history was 34.9% (15/43) in females and 17.6% (3/17) in males (p = 0.23).

**Symptom profile in urticaria patients:** overall, symptoms of chronic urticaria had been present for <1 year in 13 (21.7%), 1 - 5 years in 29 (48.3%), and >5 years in 18 (30.0%). The median duration of symptoms was thirty-six (36) months (IQR 12-96). All the patients with chronic urticaria presented with itching and wheals with symptoms lasting less than twenty-four hours. The location of itching and wheals was generalised in all patients with the hands being most frequently involved (95%, 57/60). The duration of wheals was less than 1 hour in 11 (18.3%), and 1 - 24 hours in 49 (81.7%). Remission rates based on duration of chronic urticaria were as follows: ≤1 year - 0 (0%), > 1 year - 32 (53.3%). Angioedema occurred in 28 (46.7%) patients, and the duration was up to 72 hours in 20 of these (71.4%).

A personal history of atopy was present in 9 (15.0%) and a history of asthma in 4 (6.7%) patients respectively. There was no relationship between atopy and nut allergy (Fischer´s exact p = 0.05) and also none between asthma and nut allergy (Fischer´s exact p = 0.57). Systemic symptoms were present in 27 (45%) of patients. Flushing was described as feeling blood rush to the face with associated redness in some cases. None of the patients had history or physical findings suggestive of an autoimmune disease such as recurrent fever, hair loss, joint pains, and weight loss. There was no palpably enlarged thyroid gland in all the patients and no history or clinical findings suggestive of a thyroid disease.

**Figure 2:**
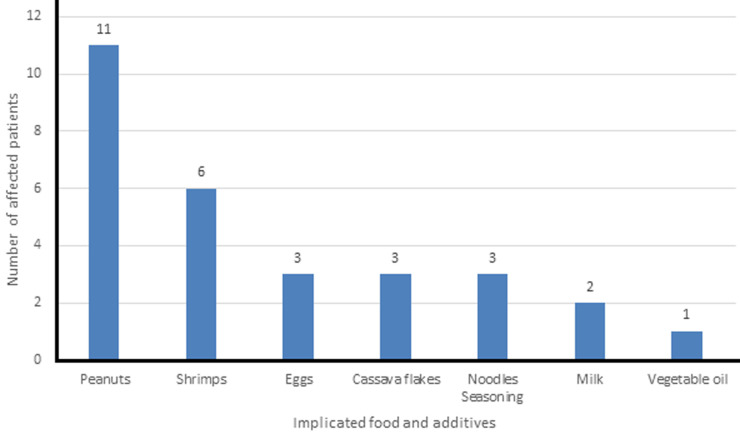
implicated food and additives

**Precipitants of chronic urticaria:** precipitants were identified in 16 (26.7 %) participants, and included food (including food additives), drugs and environmental factors (Table 1). Food was the most frequently identified precipitant. Of these, peanuts were the most common precipitant (11, 18.3%), followed by shrimps (6, 10%). Nine (15%) of the patients with precipitating factors had more than one identifiable precipitant. Similarly, 8 (13.3%) participants had a history suggestive of multiple food precipitant (Figure 2).

**Table 1:** precipitating factors in urticaria subjects

Precipitants	Frequency n=60 (%)
Identifiable precipitants	16 (26.7)
Multiple precipitants	9 (15.0)
Food and additives	14 (23.3)
Multiple food types	8 (13.3)
Exposure to areas with plants and flowers	9 (15.0)
Stressors	8 (13.3)
Psychological stress	5 (8.3)
Physical exertion	3 (5)
Drugs	4 (6.7)
NSAIDS	3 (5)
Paracetamol	1 (1.7)

NSAIDS: nonsteroidal anti-inflammatory drugs

**Figure 3:**
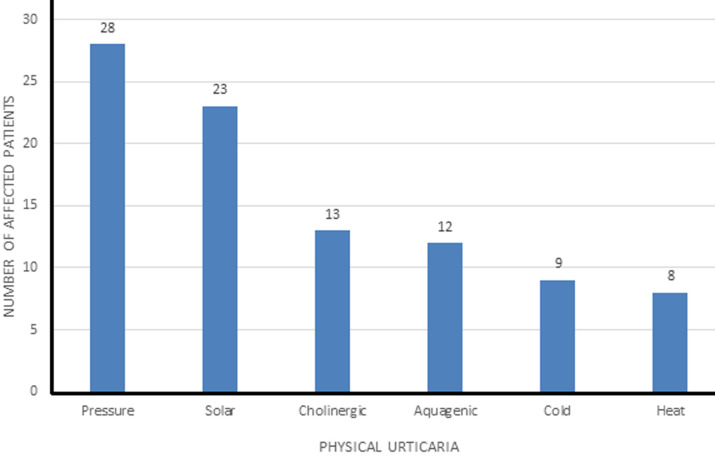
frequency of different types of self-reported physical urticaria (multiple responses of implicated physical factors in some patients)

**Figure 4:**
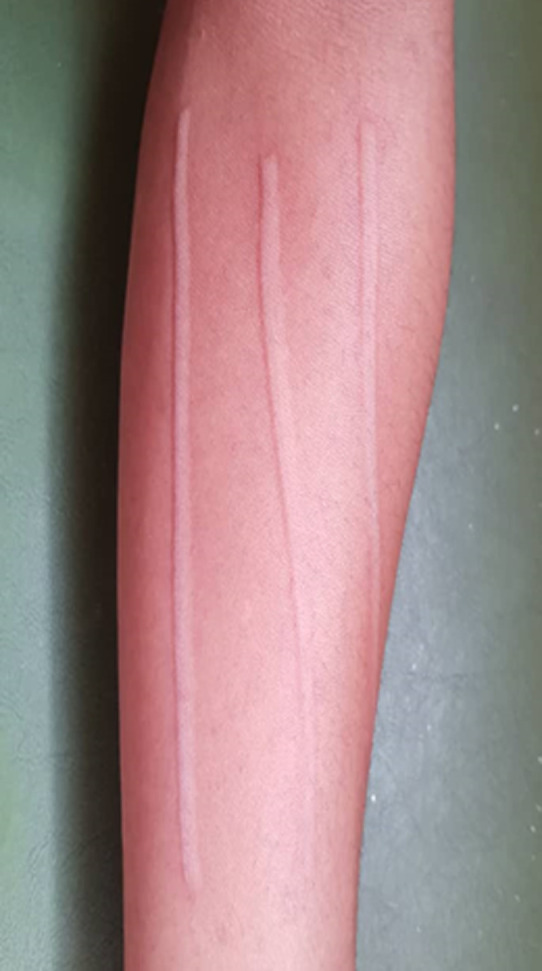
dermographism on the left forearm of a patient

**Implicated physical factors:** although eliciting tests were not carried out to assess for physical urticaria, self-declared history suggestive of physical factors as precipitants was present in 42 (70%), with pressure urticaria being the most common (28, 46.7%). History suggestive of more than one type of physical urticaria was documented in 32/42 (76.2%), with 6/42 (14.3%) having as high as three types of physical factors precipitating urticaria in them (Figure 3). Dermographism was also assessed for in both patients and controls which was positive in 37/60 (61.7%) and 14/60 (23.3%) respectively (p = 0.00) (Figure 4).

**Figure 5:**
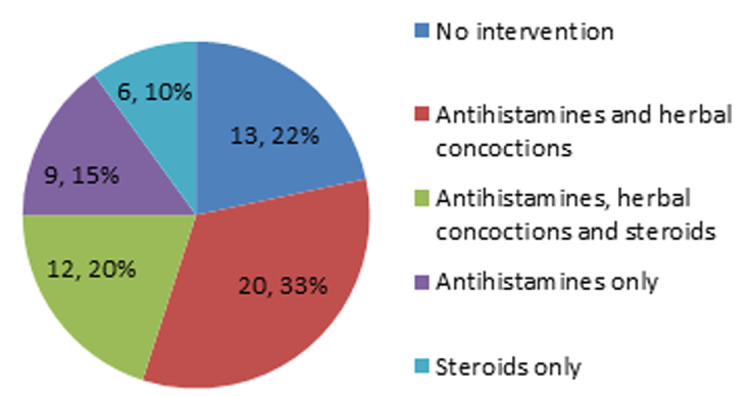
interventions used prior to presentation

**Intervention prior to presentation:** intervention had been done prior to presentation in 47 (78.3%) patients and 32 (53.3%) out of the total patients had used both pharmacological and herbal concoctions. Pharmacological intervention was prescribed by a general physician in 26 (43.3%) patients and by a pharmacist in 21 (35.0%) patients. None of the patients had seen a dermatologist prior to presentation (Figure 5).

**Hematological and serological tests:** HBsAg was positive in 4 (6.7%) cases and 1 (1.7%) of the controls (p = 0.36). Screening for hepatitis C was negative in all patients and controls. Anti-thyroid peroxidase (Anti-TPO) assay was positive in 4 (6.7%) of patients. ESR >20mm/hr was noted in 53.3% (32) of patients. All other haematological investigations were within normal limits.

Stool for ova and parasite examination was positive in 4 (6.7%) of chronic urticaria patients and 2 (3.3%) of the controls. Two patients (3.3%) had ova of *Entamoeba coli* and two (3.3%) had *Ascaris lumbricoides* ova. The two controls who were positive had ova of *Entamoeba coli*. All results compared were not statistically significant (p = 0.68). H. pylori antigen in stool was positive in 35 chronic urticaria cases (58.5%) and 43 (71.7%) of the controls (p=0.13).

## Discussion

The need for an attempt to explore the aetiology of chronic urticaria cannot be over-emphasized as the condition tends to run a chronic course and if a possible cause can be found, patients may be relieved of their symptoms. The results from the present study corroborated a great deal with findings of previous work done in this field with some differences observed in this population. More than two thirds of patients in the study had perceptions about what was causing the condition ranging from food to environmental and physical factors. Avoidance of such perceived causes however, led only to a reduction in frequency and severity of symptoms but not a complete elimination.

The demographic distribution in this study resonates with that of the previous studies that have been done on chronic urticaria [3]. The peak age of chronic urticaria was in the third decade of life for both genders which was in keeping with findings from previously done studies. The age of onset of urticaria however followed the same profile in both sexes with the peak age of onset in the third decade of life. The female preponderance may therefore be a true representation of demographic distribution or may reflect a better health seeking behaviour of women. Furthermore, females may also be more likely to have concern about skin issues and therefore present to the clinic more often than men. Family history of urticaria in first degree relatives was found in close to a third of patients in this study. Asero *et al*. reported a family history of urticaria in 4% of patients in a large study of over a thousand patients with chronic urticaria [4]. This brings to fore the possible contribution of genetic factors to chronic urticaria which have previously been reported in the literature [5].

Most of the patients in this study had symptoms between one to five years with almost a quarter of patients having symptoms of urticaria over five years showing the chronicity of the condition. The high percentage of patients with long standing duration of symptoms may be accounted for by the study site, which is a tertiary centre, hence most of the patients referred there are likely cases not responding to conventional treatments. Over two thirds of patients had tried different forms of treatment prior to presentation including pharmacological and use of herbal concoctions with no remission of symptoms. Patients that had symptoms lasting more than one year had chronic recurrent urticaria with periods of remission where they were completely symptom free, only for symptoms to recur later. The chronicity of the condition was also documented by Gaig *et al*. in a study done in Spain which noted that 8.7% of patients had symptoms between one to five years and 11.3% had symptoms lasting more than five years [6]. A prospective study involving 139 patients by Toubi *et al*. noted that urticaria had lasted more than one year in over 70% of patients and over five years in 14% of patients studied [7].

Angioedema occurred in almost half of the patients similar to findings in previous studies [6]. In fact, some patients had been having urticarial wheals for months, but only presented when the more worrisome angioedema occurred. Ferrer *et al*.documented the presence of urticaria and angioedema in 30.8% of patients studied while another study done by Toubi *et al*. noted angioedema in 40% of patients with chronic urticaria [7,8]. Precipitants were identified in almost a third of the patients in this environment and most patients reported more frequent symptoms when exposed to such precipitating factors and long periods of remission when such precipitants are avoided. Similar to some studies, food precipitants were identified in about a third of patients with nuts being the most commonly implicated food followed closely by shrimps [8]. Magerl *et al*.recognised that a pseudo-allergen free diet improved symptoms in over a third of patients with chronic spontaneous urticaria [9]. Cassava flakes commonly known as *‘garri´* is a staple food in this environment which is either taken in its raw form soaked with water at room temperature or as a meal made with hot water known as *‘eba´*. Intake of *‘garri´* precipitated urticaria in 5% of patients; it was however, noted that the patients developed symptoms only when it was taken in its raw form. Symptoms did not occur in the patients when it was taken in the solid form. This may be explained by some contaminants which get into the cassava flakes during storage which may be eliminated when hot water is added to the flakes. Another possibility for this may be explained by improper processing of the cassava flakes during initial preparation prior to being consumed with release of substances that may precipitate urticaria in susceptible individuals. This might be important when taking a history from patients in this environment as it is a commonly eaten food in Nigeria. Another interesting food additive in 5% of patients was the seasoning from a popular noodle in this environment as those patients only had more symptoms when they use the accompanying seasoning packed with the noodles; the effect of which was lost when the pack of noodles was cooked without its seasoning. This may be explained by some additives or preservatives in the seasoning but not present in the noodle itself. It is worthy of note that there may still be several other seasoning and foods that may precipitate urticaria in susceptible individuals apart from those explored in this study.

All the patients in this study had chronic spontaneous urticarial; however, a high percentage of them also had symptoms suggestive of physical urticaria with some patients having a history of more than one type of physical urticaria. Coexistence of chronic idiopathic urticaria with physical urticaria has been reported previously and occurrence of more than one type of physical urticaria in the same individual has been documented in literature [10]. Diverse results exist in literature about the prevalence of physical urticaria with prevalence as high as 35% [6,11]. It is however worthy of note that avoidance of the implicated physical factors did not lead to complete resolution of urticaria and patients still had symptoms albeit reduced after efforts were made to avoid the physical factor. The high prevalence of physical urticaria in this study can be explained by the small sample size, the homogeneity of the study area and the fact that diagnosis of physical urticaria was based on history alone without provocative tests carried out on the patients which may lead to over-reporting of physical urticaria.

Several studies conducted have suggested the causes of chronic urticaria; ranging from infections, infestations to autoimmunity and allergy [12]. There was a slight elevation in ESR with all other haematological parameters within their normal ranges in this study. This is similar to what was observed by Kulthanan *et al*. in a retrospective study of 450 patients with chronic urticaria [13]. There have been some reports of eosinophilia, basophilia and elevated white cell counts in the presence of allergic conditions and infections [14]. Association of chronic urticaria with infections have been conflicting and most studies report no significant association [15]. None of the patients in this study had any clinical or laboratory evidence suggestive of a systemic infection which is similar to what was documented in most previous studies.

In this study, controls had higher rate of infection with H. pylori using the stool antigen test, although there was no statistically observed difference. This high rate of infection rate in both controls and patients could be accounted for by the high prevalence of H. pylori in the tropics [16]. In all the patients tested for H. pylori, five had symptoms suggestive of dyspepsia although no endoscopy was done for confirmation. Association between H. pylori and chronic urticaria has been noted by some studies with improvement in symptoms after eradication of H. pylori [17]. This study found no association between H. pylori infection and chronic urticaria; however, the possibility of symptom improvement following eradication of H. pylori was not explored.

The stool for ova and parasite in this study using a highly specialised concentration method which allows for visualisation of most parasites had similar results in both patients and controls with no statistically observed difference. The presence of *Entamoeba coli* cyst which is a non-pathogenic normal commensal form of the protozoa *Entamoeba histolytica* was found in both patients and controls in addition to ova of *Ascaris lumbricoides* in two patients. This finding is similar to what has been done in most developed countries showing no association between infestations in both study groups contrary to the high prevalence reported in the general population [18].

The association of chronic urticaria and acute or chronic hepatitis has also been documented by several studies. It was particularly important to assess for this as hepatitis B and C infections have a high prevalence in the tropics [19]. Similar to most studies conducted previously, there was no significant difference in the results of hepatitis B and C screening for patients and controls in this study [20]. Presently, there is still no convincing evidence of a causal relationship between hepatitis viruses and chronic urticaria.

**Limitations:** the study was carried out in a teaching hospital and may be more representative of people with longer disease duration and severity. This may be different from what is obtainable in a community-based study. The physical factors may also have been overestimated because provocative tests were not carried out.

## Conclusion

The clinical profile of chronic urticaria here appears to be similar in Caucasians as noted in most previously documented studies. The investigated antigenic aetiologies were not found to play a contributory role. It is important to explore precipitants peculiar to this environment as this may help in abating the symptoms in affected patients. Thorough history is still the most important guiding factor in the assessment of patients with chronic urticaria as this will reduce the need for extensive investigations.

### What is known about this topic

- Several factors have been implicated in the aetiology of chronic idiopathic urticaria and the aetiology remains largely uncertain;
- A thorough clinical history is important in the management and will help reduce the need for extensive investigations;
- Varying reports about the role of infestations and viral infections like hepatitis B and C in the aetiology of chronic urticaria especially in developing countries.

### What this study adds

- This study adds to the body of knowledge about possible implicated factors in chronic urticaria in this environment as there are limited studies on that;
- Some specific precipitants peculiar to this environment were also highlighted in this study;
- Infestations and viral infections like hepatitis B and C, helicobacter pylori may not play an important role in the aetiology of chronic urticaria even in developing countries contrary to some previous reports.
